# Supplementary material for: Daily versus stat vitamin D supplementation during pregnancy; A prospective cohort study
Source: PLoS One. 2020 Apr 16;15(4):e0231590. doi: 10.1371/journal.pone.0231590 (PMC7162461; doi:10.1371/journal.pone.0231590)
Supplement: Attachment — Submitted filename: Comments on PONE-D-19-29625_R1.docx [file pone.0231590.s002.docx]

The authors have addressed most of the questions. However, some were yet to be solved.

1. Season or month of the year at the baseline should be taken into account throughout all analyses, given it is a crucial influencing factor of vitamin D (VD) level.
2. As is shown in Table 2, VD levels at the baseline and follow-up were compared among the four groups, respectively. Besides, paired t-tests were performed to examine the VD increment after VD supplementation (Table 3) within each group. However, we would like to know whether the difference of VD increment among the four groups is statistically different (especially between G2 and G3). Thus, the VD increment of each group should be compared, and a multivariate model should be employed to control the confounding of baseline VD level, and more importantly, the season (or month of the year) at the baseline, as well as other potential confounders.
3. Did the authors assess the compliance of each intervention group regarding VD supplementation?
4. In the **Statistical Analysis section**, “Fischer exact test” is supposed to be “Fisher exact test.”
