# Supplementary material for: Daily versus stat vitamin D supplementation during pregnancy; A prospective cohort study
Source: PLoS One. 2020 Apr 16;15(4):e0231590. doi: 10.1371/journal.pone.0231590 (PMC7162461; doi:10.1371/journal.pone.0231590)
Supplement: Attachment — Submitted filename: Point by Point Response.docx [file pone.0231590.s003.docx]

**RESPONSE TO REVIEWERS**

**Respected Editor,**

We have received second round of revisions/suggestions for our submitted manuscript. All the concerns and suggestions of the reviewer have been addressed and we hope that the revised version of the manuscript will satisfy the concerns of all reviewer. We have attached point-by-point response to the reviewer and revised version of manuscript is also highlighted to track the changes. Please let us know if any other changes are required in this regard. Last but not least, we are very thankful to editor and reviewers for their time and efforts to put their valuable suggestions. Indeed their recommendations made this manuscript more scientifically elegant and sound.

**REVIEWER # 1**

**Query – 1: Season or month of the year at the baseline should be taken into account throughout all analyses, given it is a crucial influencing factor of vitamin D (VD) level.**

**Response – 1:** Respected reviewer, thank you for the recommendation. The study was carried out in the colder months of the country i.e. October 2018 to April 2019 [Autumn = 22^nd^ September – 21^st^ December, Winter = 22^nd^ December – 20^th^ March, Spring = 21^st^ March - 21^st^ June]. A total of 112 patients were recruited in Autumn season, 104 in Winter and 65 in Spring. The UV index (UVI) is comparatively low in these months. The optimal UVI for the production of Vitamin D (D-UV) is greater than 3.^1,2^ However, the UVI decreases from October to December and then raises from January to April. Moreover, Chi-square test was preformed to check association of season with the VD level at the baseline; the test was insignificant (p = 0.072). With respect to your conerns, we have also highlighted the same issue in the limitation section of the manuscript.

1. Holick MF. Vitamin D Deficiency. N Engl J Med 2007; 357: 266-81.
2. Hollick MF, Chen TC. Vitamin D deficiency: a worldwide problem with health consequences. Am J Clin Nutr 2008; 87: 1080S-6S.

**Query – 2: As is shown in Table 2, VD levels at the baseline and follow-up were compared among the four groups, respectively. Besides, paired t-tests were performed to examine the VD increment after VD supplementation (Table 3) within each group. However, we would like to know whether the difference of VD increment among the four groups is statistically different (especially between G2 and G3). Thus, the VD increment of each group should be compared, and a multivariate model should be employed to control the confounding of baseline VD level, and more importantly, the season (or month of the year) at the baseline, as well as other potential confounders.**

**Response – 2:** Respected Reviewer, Thank you for the suggestion. VD increment between all the groups has been checked as per your recommendation. All groups shows that there is a significant difference between the control group and treatment cohorts (G1, G2 and G3); (p < 0.001). The treatment groups were also compared with each other and there was statistically significant difference [G1 and G2; (p < 0.001); G1 and G3; (p < 0.001)]. However, there was no statistically significant difference between G2 and G3 (p = 0.579). This information has been updated in the manuscript in the text before Table 3. To the best of our knowledge, if covariates are equally distributed between the treatment groups then confounder adjustments are not necessary during the analysis. However, we respect your suggestion and carried out regression analysis in which we adjusted the season to determine the potential contributors of vitamin D insufficiency among study participants.

**Query – 3: Did the authors assess the compliance of each intervention group regarding VD supplementation?**

**Response – 3:** Respected Reviewer, patient compliance was self-reported by patients and was assessed during the follow-up visit. All the patients in the treatment groups were compliant with their medication regimen.

**Query – 4: In the Statistical Analysis section,** **“Fischer exact test” is supposed to be “Fisher exact test.”**

**Response – 4:** Respected Reviewer, Thank you for pointing out the typo. Manuscript has been updated accordingly.

**Corresponding author: Yusra Habib Khan**
