# Supplementary material for: Daily versus stat vitamin D supplementation during pregnancy; A prospective cohort study
Source: PLoS One. 2020 Apr 16;15(4):e0231590. doi: 10.1371/journal.pone.0231590 (PMC7162461; doi:10.1371/journal.pone.0231590)
Supplement: Attachment — Submitted filename: Point by Point Response.docx [file pone.0231590.s004.docx]

**RESPONSE TO REVIEWERS**

**Respected Editor,**

We have received third round of minor revisions/suggestions for our submitted manuscript. All the concerns and suggestions of the reviewer have been addressed and we hope that the revised version of the manuscript will satisfy the concerns of the reviewer. We have attached point-by-point response to the reviewer and revised version of manuscript is also highlighted to track the changes. Please let us know if any other changes are required in this regard. Last but not least, we are very thankful to editor and reviewers for their time and efforts to put their valuable suggestions. Indeed their recommendations made this manuscript more scientifically elegant and sound.

**REVIEWER # 1**

**Query – 1: 1. In the abstract, please provide the VDD frequency after intervention.**

**Response – 1:** Respected Reviewer, VDD frequency has been added in the abstract section.

**Query – 2: 2. Conclusion has better be simplified.**

**Response – 2:** Respected Reviewer, as per your suggestion we have simplied the conclusion section of the manuscript. I hope that current version of conclusion will satisfy the concern.

**Query – 3: While the effect of VD supplement was compared between groups, the VD influence factors such as sun-exposure time, Use of sunblock and baseline VD level must be adjusted. This part is the main result for evaluating the effect of intervention. Then add this part to manuscript.**

**Response – 3:** Respected Reviewer, the confounding factors has been adjusted. This resulted in addition of another table controlling the impact of covariates on outcome variable. We have also modified the results according to the new table. We really hope that the current version of manuscript will satisfy your concern in this regard.

At the end, are are greatly thankful for your time and interest in our research . Indeed, your suggestions were quite valuable for us. Our believe on peer review is more strenghthened amid healthy discussion pertaining to current research.

**Corresponding author: Yusra Habib Khan**
